# The cost of cancer care is not related to its outcomes

**DOI:** 10.3332/ecancer.2016.687

**Published:** 2016-10-28

**Authors:** Guy Storme, S Dhaese, D Corens, Mark De Ridder

**Affiliations:** 1 Department of Radiation Oncology, UZ Brussel, Belgium; 2 Financial Department, UZ Brussel, Belgium

**Keywords:** cost-effectiveness, surgery, radiotherapy, systemic treatment

## Abstract

Solid tumours make up 90% of all proliferative diseases and the main action for cure remains surgery, removing the visible tumour as well as the surrounding tissue. Radiotherapy is an added value for eliminating local microscopic as well as regional disease. Systemic treatment has a small impact on the outcome but has a cost, which is as much as all the other actions such as diagnostic tools and treatments.

## Background and introduction

The cost of health care is still increasing and the top of the bill is still not in view. New treatments, mainly that of pharmaceutical agents, are exploding [1] and are only effective at a very low level without a clear impact on the population [2].

## Methods

Hospital bills of individual incoming patients (n = 637) at the Oncologic Centre UZ Brussel between 01 January 2006 and 30 June 2006 were individually analysed according to the different actions described and summed up to avoid too much detail: consultation, surgery, hospitalisation, day hospital, pharma, medical material, radiology, anatomopathology, clinical biology, radiotherapy, nuclear medicine.

All bills were followed for five years or until the patients died, taking also into account the cost of relapses and palliation. Individual costs such as travelling, work loss etc. were not taken into account.

## Results

Table 1 shows the individual described costs in Euros of the summed up activities. The main cost is pharmaceutical agents and together with the administration of them (D Hosp) it makes up 49.7% of the total cost. Surgery being the most important curable factor, at least for solid tumours, is only 4.9% and radiotherapy 9.7%. Diagnostic procedures covering radiology, anatomopathology (which is the confirmation of the exact tumour origin), clinical biology and nuclear medicine makes up 17% of the total bill. The initial cost (first year) is 53.4% of the total five-year cost and that for the fifth year is only 8.4%, the latter corresponds mostly to palliative care for relapsing patients. Our patient distribution in order of localisation and percentage is: breast 22%, brain (including metastatic locations 16%; lung 9%, prostate 9%, rectum 6%, head and neck 5%, skin 3%, … The work-up as well as the treatment was done according to international guidelines. The outcomes of our data in breast-[4–6], rectum-[7–8], head and neck-[9], lung-[10], and prostate cancer [11–12] were published in several peer reviewed papers and also at the European collaborative level [7, 13].

## Discussion

The cost of health care is rapidly increasing, mainly in the development of new drugs which have increasingly been focused on ‘personalised’ medicine in recent years [1]. In the period analysed, there was no major new drug reimbursed by social security and this analysis is certainly no representative of the present day increasing cost mainly because those drugs use immunomodulatory activity [17]. The positive impact of systemic treatment is limited [2]. As for example say in breast cancer,when we observe patients with taxanes and herceptine, accepted as part of routine treatment, in node positive patients on top of hormone treatment like oestrogen positive patients and also with agressive treatments in triple negative patients from 2000 on, the impact on the population is not that obvious at five years (90.1–91%) [3]. If we evaluate precision medicine thus far in many patients (18,000) who have undergone sequencing in the past decade, the number of reported cases are rare and only a few showed a complete response [14]. Moreover, the good responders were already good responders to chemotherapy.

The only proofs of the bonus of ‘precision or targeted’ medicine are randomised trials and the SHIVA trial. We see treating patients according to their identified mutations versus selected treatment by the physician showed no difference in progression-free survival (PFS) (2.3 veruss 2.0 months) [15]. Even if we have some long living patients like in melanoma, we need to wait longer for the impact to be seen on five year survival.

Recently the US Food Drug Administration (FDA) approved a treatment for melanoma where it shrunk 60% of tumours in a clinical trial The company charged 141,000 US dollars for the first 12 weeks and 256,000 US dollars for a year! A study of the National Bureau of Economic Research in 2015 found drug prices had increased by 10% every year between 1995 and 2013, an annual increase of around 8500 US dollars, which resulted in an average cost for one extra year of life of 54.000 US dollars in 1995, increasing to 139.000 US dollars in 2005, and 207,000 in 2013 [16].

However, the major outcome bonus is related to shift in stage at diagnosis to screening (50%shifted from SII to SI in breast cancer between 1989 and 1999 SEER DATA). The same is observed for colorectal cancer and prostate cancer: since 2000 no bonus has been observed (data not shown). In non-small cell lung cancer NSCLC, a bonus of 3% has been observed since 2000, but this is perhaps because of better diagnostic tools such as PET-CT scan which is now mandatory before starting treatment. Previously, it was described that the bonus of cytotoxic chemotherapy was about 2.1–2.3% [2] and we are now confronted with new molecules at astronomic amounts for a single treatment, of which the bonus is difficult to evaluate on population outcome so far [1,16].

For most people arguing for new drugs even by trials outcome, it should be worthwhile to read the paper by L Saltz: ‘The Value of Considering Cost, and the Cost of Not Considering Value’ [16] and his conclusion ‘We need to understand, and we must help our patients, our partners in industry, and our elected officials to understand, the meaning and importance of value, and that there must be upper limits to cost. We must recognise and then work to remove the perverse incentives that impede the healthy functioning of the cancer drug market’.

We here end with Prasad’s recent thought on precision medicine: ‘We can ask if rhetoric so far outpaces the reality that we risk fooling even ourselves’ [18].

## Conclusions

Cost effectiveness in cancer care is inversely proportional to outcome, mainly in solid tumours, which from 2000 on has had nearly no impact on the population outcome. Politicians should take this into account when reimbursing health care for cancer.

## Conflicts of interest

No conflicts of interest.

## Figures and Tables

**Table 1.** Total five year cost (In Euros) for individual bills by medical activity.

Results	2006	2007	2008	2009	2010	Total Five Y
SX	285,565	51,845	32,841	25,459	24,777	393,486
Consult	83,375	49,741	32,898	24,806	22,025	212,845
Hospit	620,447	147,694	112,607	103,364	78,213	1,087,505
D Hosp	147,770	794,36	51,337	27,977	33,570	340,090
Pharma	1,876,795	690,390	478,294	295,980	338,022	3,679,481
MedMat	129,313	32,702	23,155	21,458	16,030	222,658
Radiol	238,762	111,347	100,670	86,639	74,459	611,877
Lab AP	140,005	22,921	18,353	13,436	14,075	208,789
ClinBiol	116,762	46,379	27,473	28,510	22,438	241,563
Radioth	565,927	81,115	56,014	50,693	33,610	787,359
NuclMed	140,146	63,406	42,329	33,907	26,602	30,6391
Total	4,317,868	1,376,976	975,971	712,229	683,820	8,092,043

**List of abbreviations used**

SX: surgery, Consult: consultation, Hospit: hospitalisation, MedMat: medical material, Radiol: radiology, Lab AP: laboratory anatomopathology, ClinBiol: clinical biology, Radioth: radiotherapy, NuclMed: nuclear medecine, Pharma: pharmaceutical agents.
